# Supplementary material for: Observing shifts in phenology of tropical flowering plants
Source: PLoS One. 2026 Feb 25;21(2):e0342105. doi: 10.1371/journal.pone.0342105 (PMC12935240; doi:10.1371/journal.pone.0342105)
Supplement: S1 File — (DOCX) [file pone.0342105.s001.docx]

Supplemental:

**Data Subsets.** Initial download of all angiosperms across all locations resulted in a list consisting of 2459 species (not shown). From this initial list, we manually reviewed photos of digitized specimens to determine presence or absence of flowering.

From this dataset of 2459 species, we then constructed six datasets considered final for purposes of downstream exploration, following implementation of four criteria (Table S1) for inclusion of a given species in each matrix: (1) maximum number of months flowering, (2) minimum number of herbarium specimens, (3) minimum span of years collected, and (4) maximum number of specimens collected in a single day (Table S1).

Table S1. Six datasets were constructed by implementing four different criteria that spanned four different parameters: (1) maximum number of months flowering, (2) minimum number of herbarium specimens per species, (3) minimum span of years collected in the flowering phenophase, and (4) maximum number of specimens collected in a single day.

| **Dataset** | **Months flowering** | **# Specimens** | **Year span** | **Specimens per day** | **# Species meeting criteria** |
| --- | --- | --- | --- | --- | --- |
| 1 | <4 | >20 | >29 | Not Applicable | 33 |
| 2 | <3 | >20 | >29 | Not Applicable | 16 |
| 3 | <4 | >50 | >29 | Not Applicable | 26 |
| 4 | <4 | >20 | On or after 1960 | Not Applicable | 32 |
| 5 | <4 | >20 | Before 1960 | Not Applicable | 11 |
| 6 | <4 | >20 | >29 | Max 1 per day | 33 |

The summarized results for each of the six criteria are below (Table S2).

Table S2. Averages pulled from each of the six datasets. Dataset number, average absolute slope, SD, number of species with positive slope (indicating flowering occurring later in the year than historic records), number of species with negative slope (indicating flowering occurring earlier in the year than historic records), average change in flowering date per decade and number of species that met the dataset criteria (Table S1).

| **Dataset** | **Slope** | **SD** | **Positive slope** | **Negative slope** | **Days per decade** | **# species** |
| --- | --- | --- | --- | --- | --- | --- |
| 1 | 0.028 | 0.04 | 23 | 10 | 2.03 | 33 |
| 2 | 0.033 | 0.015 | 11 | 5 | 1.63 | 16 |
| 3 | 0.026 | 0.016 | 17 | 7 | 1.47 | 26 |
| 4 | 0.036 | 0.02 | 20 | 12 | 2.35 | 32 |
| 5 | 0.043 | 0.028 | 6 | 4 | 2.14 | 10 |
| 6 | 0.021 | 0.032 | 23 | 9 | 1.85 | 33 |

**Impacts of implementation of dataset criteria**. In this study, we explored whether and how four different criteria involved in the construction of (six) different datasets impacted resultant patterns in tropical plant phenological shifts. Specifically, we considered the maximum number of total months flowering, the minimum number of herbarium specimens per species, the minimum span of years a species was collected in the flowering phenophase, and the maximum number of specimens (of a given species) collected from a single site in a single day (Table S1 ;Table S2; Fig S1). We found minimal variation in resultant change in flowering date across the (six) datasets. The largest variance from the overall dataset was in collections made after 1960, which had a marginally greater change in flowering date (2.35 days per decade) compared to the overall average (2.04 days per decade).

We considered whether flowering duration had an impact on resultant days shifted (Chen *et al.,* 2020; Austin *et al.,* 2024) (Table S1 ;Table S2; Fig S2). Under the assumption that measures in flowering phenology are only valid for organisms that (1) only flower once per year and (2) have relatively short, cyclical flowering events, we aimed to provide an analysis of how flowering period length affected estimated flowering shift magnitude. Our analyses showed that shifts in number of days were comparable between analyses that considered whether flowering duration was less than 4 months vs. flowering duration less than 3 months) (Table S2). Future work should continue to explore this complex topic, particularly in portions of the planet where longer flowering periods are common, to assess whether our results are likely to be widespread, as others have found.

We then considered whether specimen quantity had an impact on resultant days shifted (Table S1 ;Table S2; Fig S3), as it is understood that sample sizes affect the estimation of distribution parameters (Bertin, 2015; Calinger, Queenborough & Curtis, 2013; [Gallagher, Hughes & Leishman, 2009](https://pmc.ncbi.nlm.nih.gov/articles/PMC5888139/#ref-20)). We specifically asked whether analysis that utilized a minimum of 20 specimens yielded different results to analysis that utilized a minimum of 50 specimens. Our results showed that differences between the two types of analyses were negligible (Table S2).

Next, we considered whether date range (i.e., very early historical specimens pre-dating 1960 vs. those collected on or after 1960) had an impact on resultant days shifted as a function of differing impacts of anthropogenic climate change over the last 150 years. Specifically, we predicted that specimens collected before 1960 would demonstrate lesser days of change than specimens collected after 1960 because anthropogenic impacts on climate have been more severe since 1960 (Kharouba *er al.,* 2018; Morisette *et al.,* 2009; Nemani *et al.,* 2003). We found that, in contrast to this prediction, shifts in number of days were comparable between both datasets, i.e., before 1960 and those on or after 1960 (Table S2, Figs S2 & S3). Our results are therefore unlikely being artificially inflated by early and infrequent collection efforts, at least not in a manner that would dramatically skew our interpretations of the data.

Finally, we repeated analyses by including only one specimen per location per day in an attempt to reduce biases imparted by species that have dates with high collection rates and dates of low collection rates (Jones & Daehler, 2018; [Daru, Van der Bank & Davies, 2017](https://www.ncbi.nlm.nih.gov/pmc/articles/PMC5888139/#ref-8); [Primack *et al.,* 2004](https://www.ncbi.nlm.nih.gov/pmc/articles/PMC5888139/#ref-51); [Bertin, 2015](https://www.ncbi.nlm.nih.gov/pmc/articles/PMC5888139/#ref-1); [Robbirt *et al.,* 2011](https://www.ncbi.nlm.nih.gov/pmc/articles/PMC5888139/#ref-57); [Davis *et al.,* 2015](https://www.ncbi.nlm.nih.gov/pmc/articles/PMC5888139/#ref-9)). We found that this reduction resulted in an average overall decrease in number of days shifted per decade, from 2.0 to 1.8 across all 33 species, which was non-significant in our analyses (Table S2, Fig S4). We attribute this non-significant difference to ample collection efforts across time that work to negate the bias introduced by dates with large collection efforts (Jones & Daehler, 2018; [Daru, Van der Bank & Davies, 2017](https://www.ncbi.nlm.nih.gov/pmc/articles/PMC5888139/#ref-8); [Primack *et al.,* 2004](https://www.ncbi.nlm.nih.gov/pmc/articles/PMC5888139/#ref-51); [Bertin, 2015](https://www.ncbi.nlm.nih.gov/pmc/articles/PMC5888139/#ref-1); [Robbirt *et al.,* 2011](https://www.ncbi.nlm.nih.gov/pmc/articles/PMC5888139/#ref-57); [Davis *et al.,* 2015](https://www.ncbi.nlm.nih.gov/pmc/articles/PMC5888139/#ref-9)). Additional research that utilizes data from locations with less frequent collection efforts may find that number of specimens collected in a given day may influence resultant patterns (Jones & Daehler, 2018; [Daru, Van der Bank & Davies, 2017](https://www.ncbi.nlm.nih.gov/pmc/articles/PMC5888139/#ref-8); [Primack *et al.,* 2004](https://www.ncbi.nlm.nih.gov/pmc/articles/PMC5888139/#ref-51); [Bertin, 2015](https://www.ncbi.nlm.nih.gov/pmc/articles/PMC5888139/#ref-1); [Robbirt *et al.,* 2011](https://www.ncbi.nlm.nih.gov/pmc/articles/PMC5888139/#ref-57); [Davis *et al.,* 2015](https://www.ncbi.nlm.nih.gov/pmc/articles/PMC5888139/#ref-9)).

Fig S1. Circular Slope and circular standard deviation of species that flower for 3 months or less, with a minimum of 20 specimens, spanning a minimum of 29 years (Dataset 2). Species arranged by location.

Fig S2 ΔDOY/year of species that flower for 3 months or less, with a minimum of 20 specimens, spanning a minimum of 29 years (Dataset 2). Species arranged by location.

Fig S3. Circular Slope and circular standard deviation of species that flower for 4 months or less, with a minimum of 50 specimens, spanning a minimum of 29 years (Dataset 3). Species arranged by location.

Fig S4. ΔDOY/year of species that flower for 4 months or less, with a minimum of 50 specimens, spanning a minimum of 29 years (Dataset 3). Species arranged by location.

Fig S5. Circular Slope and circular standard deviation of species that flower for 4 months or less, with a minimum of 20 specimens, only including specimens from 1960 or later (Dataset 4). Species arranged by location.

Fig S6. ΔDOY/year of species that flower for 4 months or less, with a minimum of 20 specimens, only including specimens from 1960 or later (Dataset 4). Species arranged by location.

Fig S7. Circular Slope and circular standard deviation of species that flower for 4 months or less, with a minimum of 20 specimens, Only including specimens from before 1960 (Dataset 5). Species arranged by location.

Fig S8. ΔDOY/year of species that flower for 4 months or less, with a minimum of 20 specimens, Only including specimens from before 1960 (Dataset 5). Species arranged by location.

Fig S9. Circular Slope and circular standard deviation of species that flower for 4 months or less, with a minimum of 20 specimens, spanning a minimum of 29 years - Only including one specimen per species per day (Dataset 6). Species arranged by location.

Fig S10 ΔDOY/year of species that flower for 4 months or less, with a minimum of 20 specimens, spanning a minimum of 29 years - Only including one specimen per species per day (Dataset 6). Species arranged by location.
