# Supplementary material for: Observing shifts in phenology of tropical flowering plants
Source: PLoS One. 2026 Feb 25;21(2):e0342105. doi: 10.1371/journal.pone.0342105 (PMC12935240; doi:10.1371/journal.pone.0342105)
Supplement: S1 Table — (DOCX) [file pone.0342105.s002.docx]

| Location | Species | Family | Slope | SD | Days per decade | # Months flowering | # Years | # Specimens |
| --- | --- | --- | --- | --- | --- | --- | --- | --- |
| INPA Reserves, Brazil | *Porcelia ponderosa* Rusby | Annonaceae | 0.061 | 0.018 | 2.534 | 2 | 107 | 67 |
| INPA Reserves, Brazil | *Peltogyne pauciflora* Benth*.* | Fabaceae | -0.012 | 0.019 | 0.544 | 4 | 184 | 263 |
| INPA Reserves, Brazil | *Peltogyne recifensis* Ducke | Fabaceae | 0.27 | 0.040 | 14.118 | 4 | 57 | 37 |
| INPA Reserves, Brazil | *Ceiba erianthos* (Cav.) K.Schum. | Malvaceae | 0.022 | 0.014 | 0.855 | 4 | 192 | 352 |
| INPA Reserves, Brazil | *Ceiba jasminodora* (A.St.-Hil.) K.Schum*.* | Malvaceae | 0.028 | 0.010 | 2.698 | 1 | 52 | 27 |
| INPA Reserves, Brazil | *Ceiba schottii* Britten & Baker f. | Malvaceae | 0.026 | 0.0232 | 3.135 | 4 | 71 | 138 |
| INPA Reserves, Brazil | *Ceiba trischistandra* (A.Gray) Bakh. | Malvaceae | 0.057 | 0.035 | 4.280 | 3 | 51 | 45 |
| Tropenbos International, Bolivia | *Bougainvillea modesta* Heimerl | Nyctaginaceae | 0.069 | 0.012 | 1.877 | 2 | 116 | 60 |
| Tropenbos International, Bolivia | *Bougainvillea stipitata* Griseb*.* | Nyctaginaceae | 0.024 | 0.022 | 0.674 | 4 | 146 | 211 |
| Catimbau National Park, Brazil | *Aeschynomene martii* Benth*.* | Fabaceae | 0.015 | 0.017 | 1.201 | 4 | 113 | 218 |
| Catimbau National Park, Brazil | *Terminalia fagifolia* Mart*.* | Combretaceae | 0.013 | 0.0057 | 0.926 | 2 | 130 | 576 |
| Catimbau National Park, Brazil | *Caesalpinia pluviosa* DC*.* | Fabaceae | -0.0020 | 0.0209 | 0.0791 | 4 | 187 | 170 |
| Catimbau National Park, Brazil | *Mimosa acutistipula* (Mart.) Benth. | Fabaceae | 0.019 | 0.00932 | 1.115 | 4 | 186 | 620 |
| Catimbau National Park, Brazil | *Barnebya harleyi* W.R.Anderson & Gates | Malpighiaceae | 0.074 | 0.021 | 5.847 | 3 | 51 | 138 |
| Catimbau National Park, Brazil | *Pseudobombax parvifolium* Carv.-Sobr. & L.P.Queiroz | Malvaceae | -0.011 | 0.011 | 1.706 | 1 | 29 | 21 |
| Cocha Cashu, Peru | *Dioscorea bulbifera* L. | Dioscoreaceae | 0.0050 | 0.0066 | 0.1737 | 4 | 229 | ### |
| Bia National Park, Ghana | *Aeschynomene indica* L. | Fabaceae | -0.026 | 0.0087 | 0.791 | 2 | 217 | 542 |
| Bia National Park, Ghana | *Annona glauca* Schumach. & Thonn*.* | Annonaceae | -0.0021 | 0.021 | 0.186 | 3 | 106 | 85 |
| Bia National Park, Ghana | *Alafia barteri* Oliv*.* | Apocynaceae | 0.020 | 0.019 | 0.71 | 1 | 103 | 35 |
| Bia National Park, Ghana | *Landolphia micrantha* (A.Chev.) Pichon | Apocynaceae | -0.028 | 0.020 | 1.541 | 4 | 116 | 144 |
| Bia National Park, Ghana | *Dracaena phrynioides* Hook. | Asparagaceae | 0.044 | 0.031 | 1.768 | 4 | 156 | 79 |
| Bia National Park, Ghana | *Combretum acutum* M.A.Lawson | Combretaceae | 0.055 | 0.031 | 3.374 | 4 | 83 | 91 |
| Bia National Park, Ghana | *Terminalia laxiflora* Engl*.* | Combretaceae | -0.0043 | 0.017 | 2.537 | 4 | 145 | 245 |
| Bia National Park, Ghana | *Tetrorchidium didymostemon* (Baill.) Pax & K.Hoffm. | Euphorbiaceae | 0.0074 | 0.0070 | 10.656 | 1 | 121 | 101 |
| Bia National Park, Ghana | *Crotalaria mortonii* Hepper | Fabaceae | -0.070 | 0.039 | 4.083 | 4 | 42 | 20 |
| Bia National Park, Ghana | *Stylosanthes erecta* P.Beauv. | Fabaceae | 0.0075 | 0.021 | 3.566 | 3 | 179 | 146 |
| Bia National Park, Ghana | *Marantochloa leucantha* (K. Schum.) | Marantaceae | 0.027 | 0.017 | 1.25 | 4 | 132 | 305 |
| Bia National Park, Ghana | *Triclisia subcordata* Oliv*.* | Menispermaceae | 0.11 | 0.023 | 50.619 | 2 | 141 | 25 |
| Bia National Park, Ghana | *Tricalysia pallens* Hiern*.* | Rubiaceae | 0.0082 | 0.0087 | 9.379 | 3 | 164 | 590 |
| Bia National Park, Ghana | *Vangueriella nigerica (*Robyns) Verdc. | Rubiaceae | 0.090 | 0.022 | 5.273 | 4 | 104 | 96 |
| Southern Guinea Savanna Research Station, Guinea | *Diospyros lotus* L. | Ebenaceae | -0.022 | 0.0070 | 0.875 | 3 | 187 | 810 |
| Jatun Sacha, Ecuador | *Rudgea crassipetiolata Zappi & E.Lucas* | Rubiaceae | -0.00030 | 0.013 | 0.0369 | 1 | 46 | 33 |
| Isthmus of Kra, Thailand/ Myanmar | *Nymphoides aurantiaca* (Dalzell) Kuntze | Menyanthaceae | 0.031 | 0.012 | 1.643 | 4 | 152 | 361 |
