# Supplementary figures and images for: Observing shifts in phenology of tropical flowering plants
Source: PLoS One. 2026 Feb 25;21(2):e0342105. doi: 10.1371/journal.pone.0342105 (PMC12935240; doi:10.1371/journal.pone.0342105)

# Circular Slope for Species Flowering for 3 Months or Less

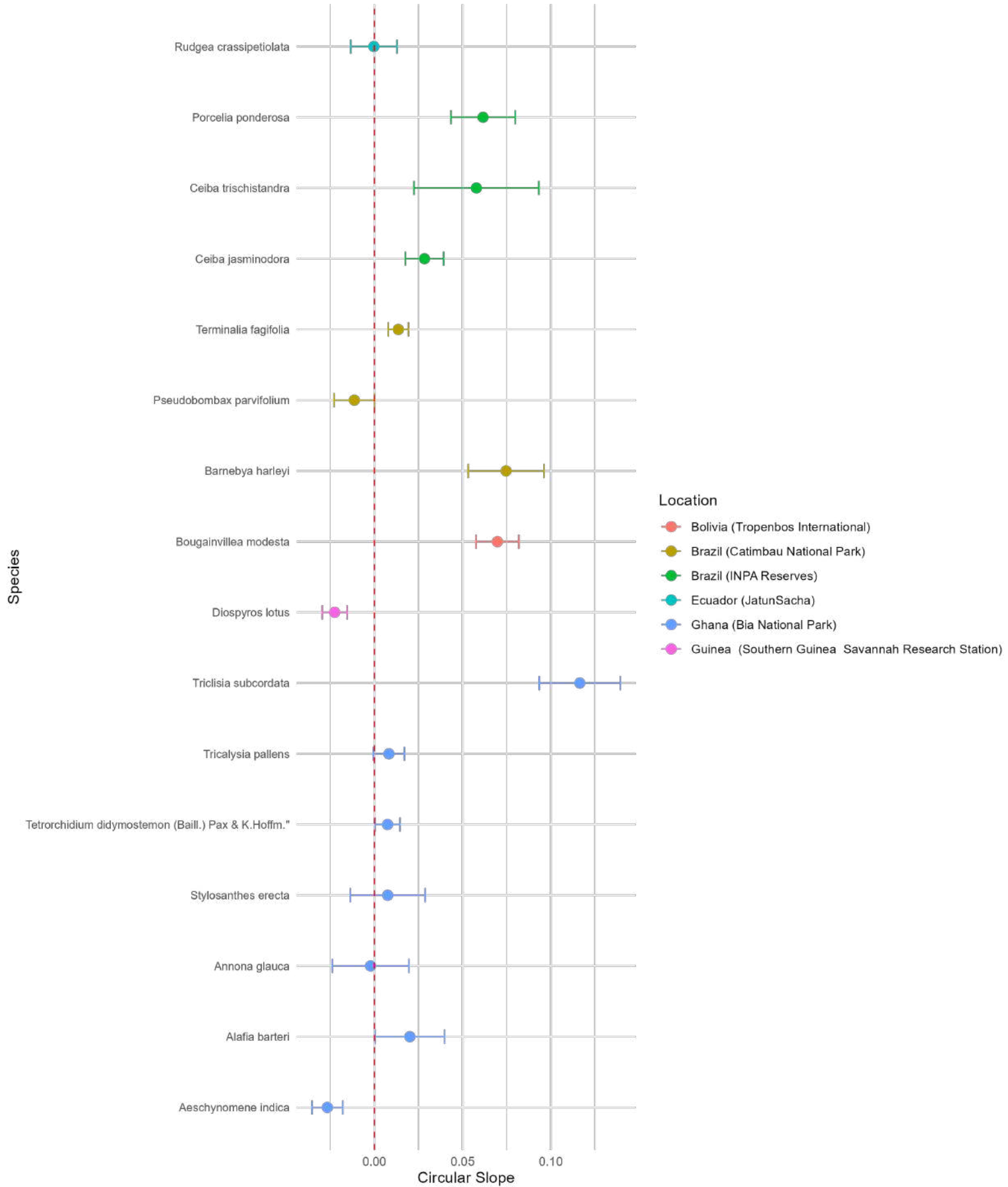

Supplement: S1 Fig — Species arranged by location. (PDF) [file pone.0342105.s003.pdf]

# ΔDOY/year for Species Flowering for 3 Months or Less

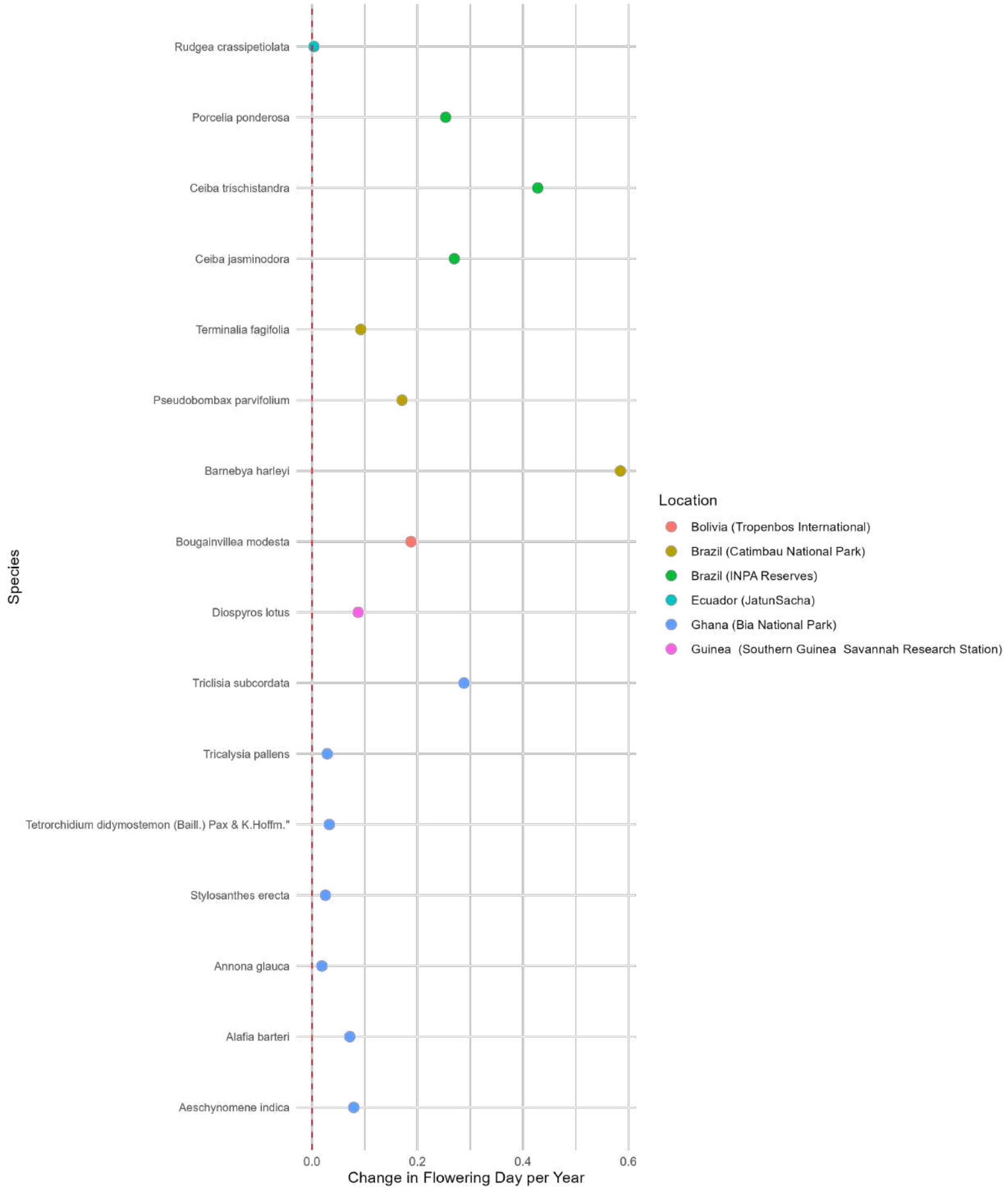

Supplement: S2 Fig — Species arranged by location. (PDF) [file pone.0342105.s004.pdf]

Circular Slope for Species with a Minimum of 50 Specimens

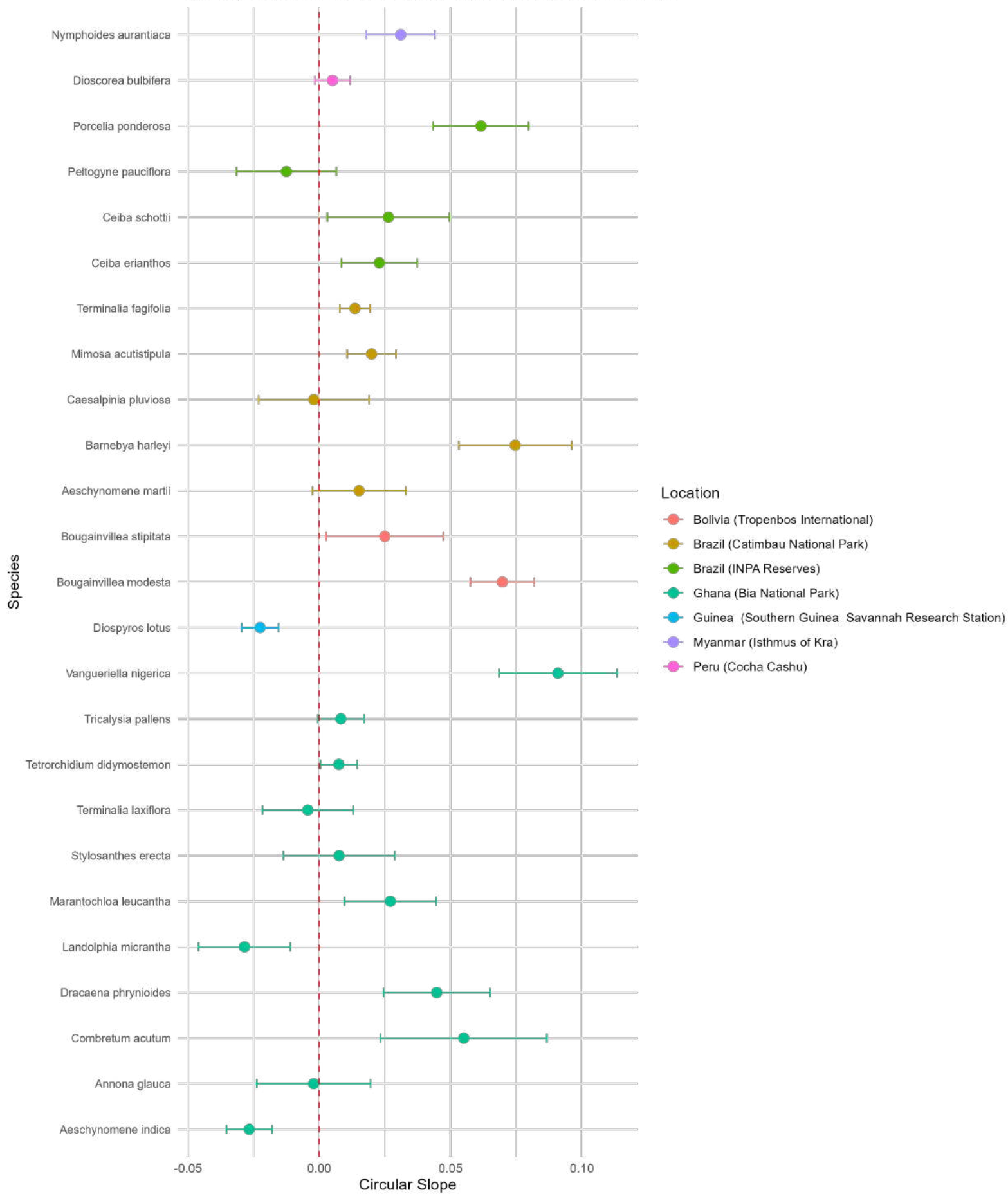

Supplement: S3 Fig — Species arranged by location. (PDF) [file pone.0342105.s005.pdf]

$\Delta$ DOY/year for Species with a Minimum of 50 Specimens

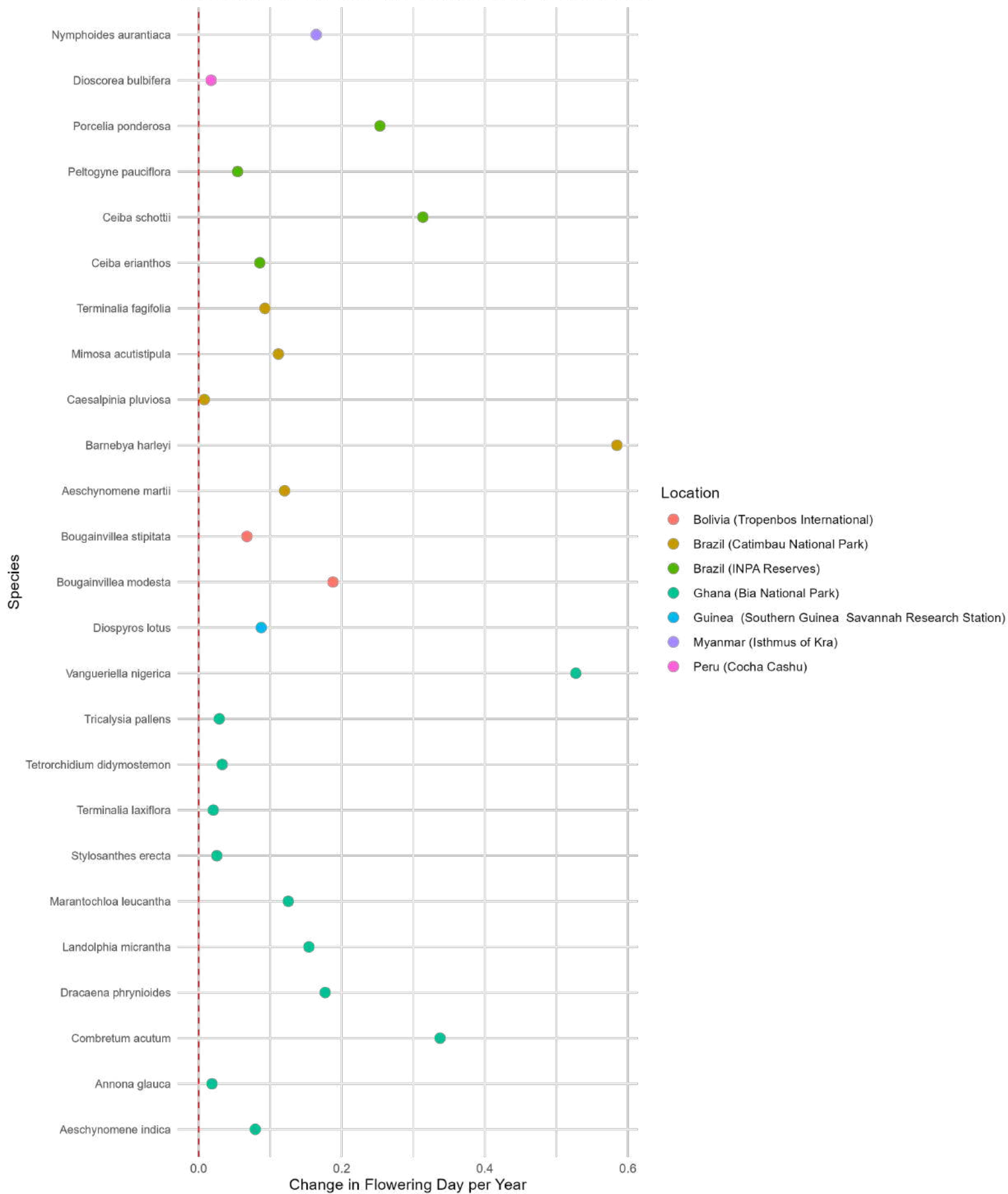

Supplement: S4 Fig — Species arranged by location. (PDF) [file pone.0342105.s006.pdf]

Circular Slope for Specimens after 1960

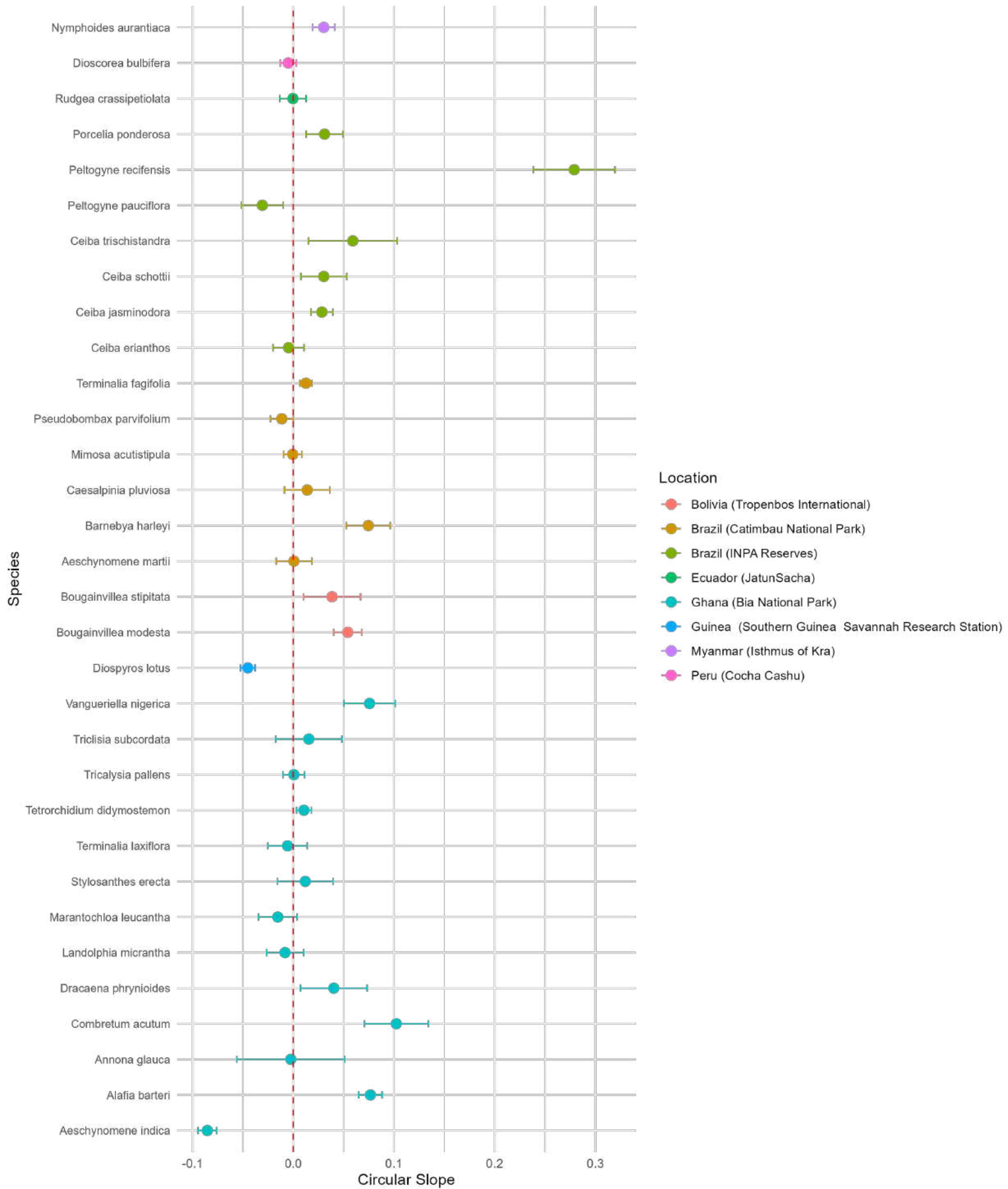

Supplement: S5 Fig — Species arranged by location. (PDF) [file pone.0342105.s007.pdf]

$\Delta$ DOY/year for Specimens after 1960

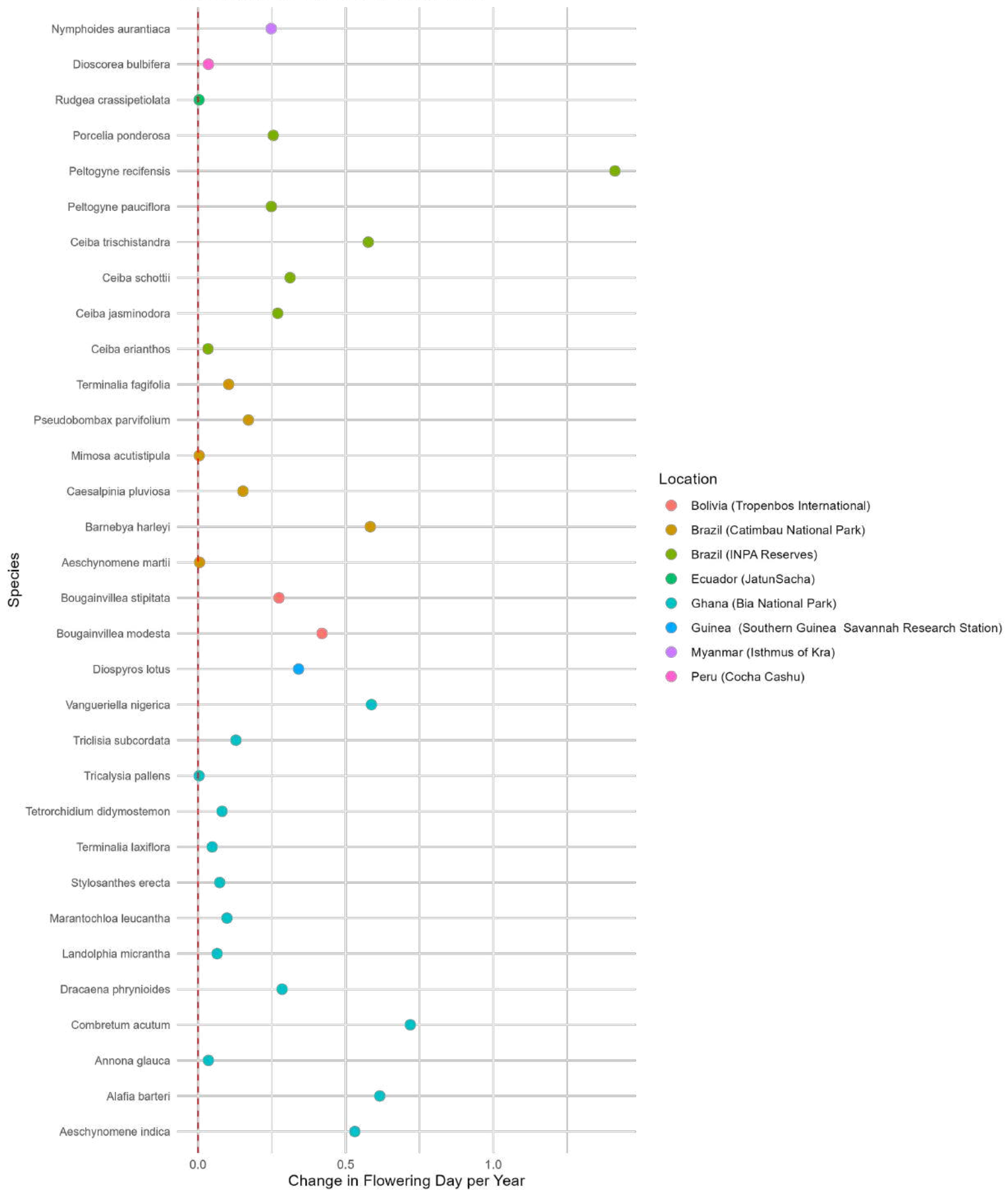

Supplement: S6 Fig — Species arranged by location. (PDF) [file pone.0342105.s008.pdf]

Circular Slope for Specimens before 1960

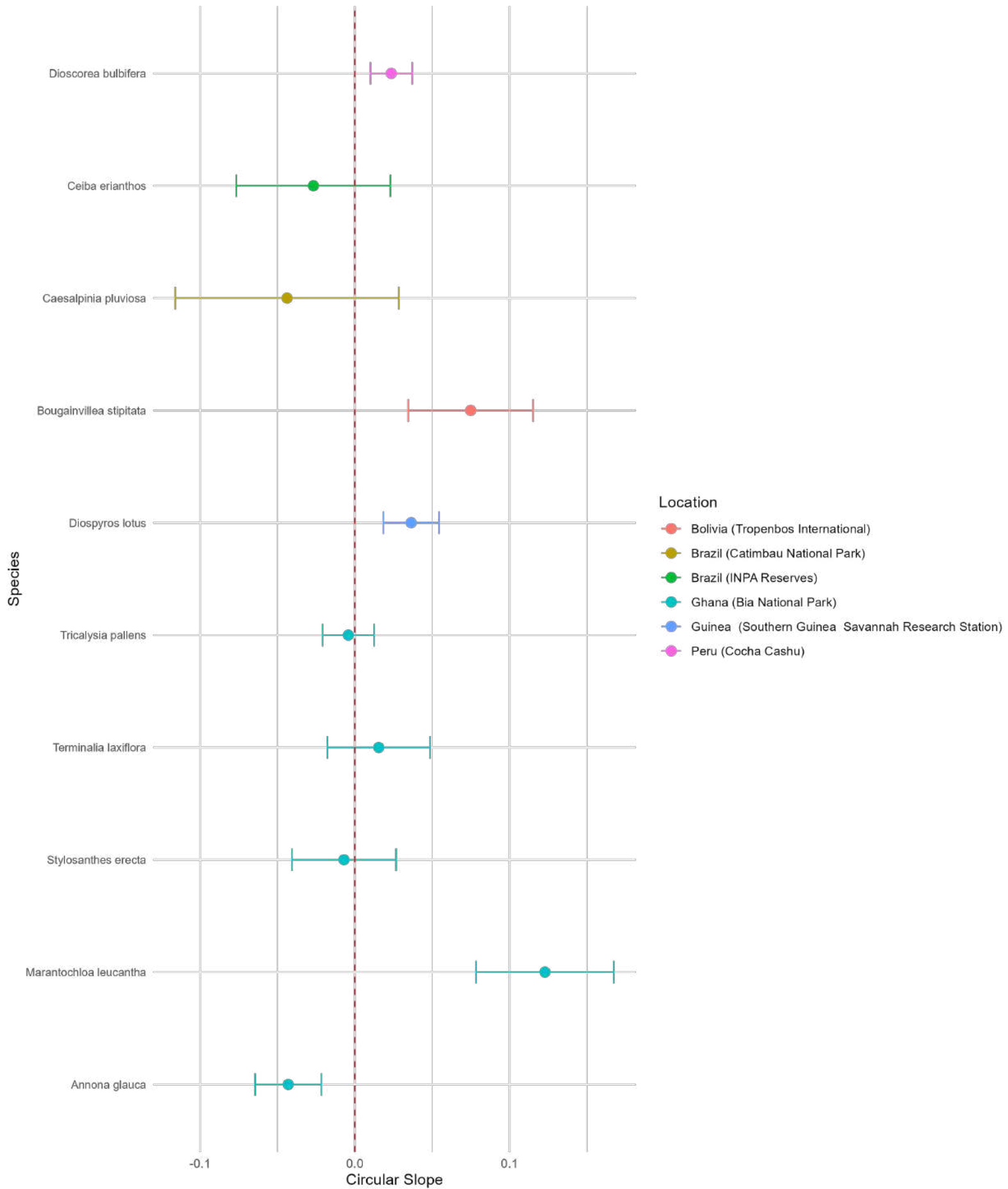

Supplement: S7 Fig — Species arranged by location. (PDF) [file pone.0342105.s009.pdf]

# $\Delta$ DOY/year for Specimens before 1960

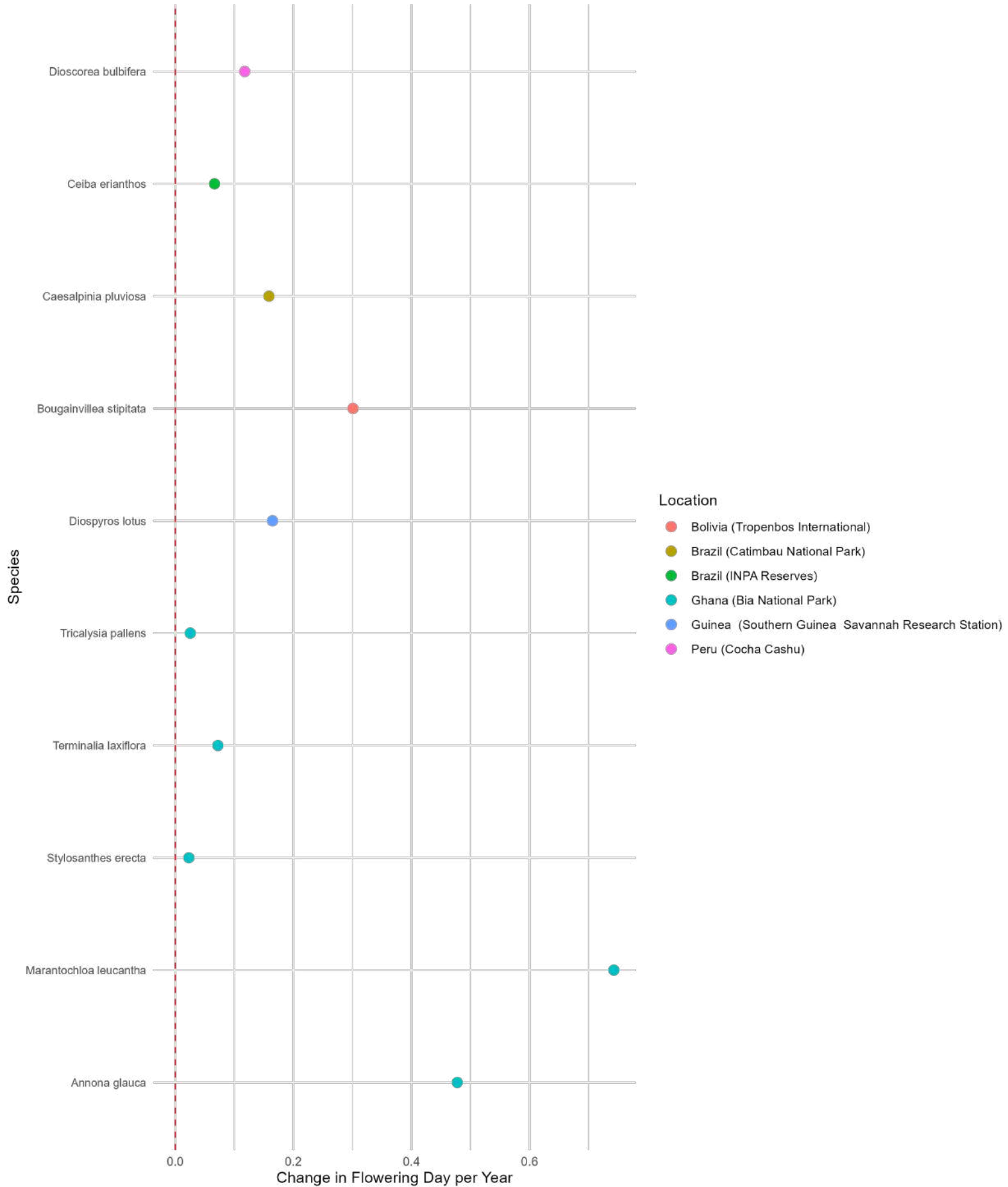

Supplement: S8 Fig — Species arranged by location. (PDF) [file pone.0342105.s010.pdf]

Circular Slope for One Specimen Per Day

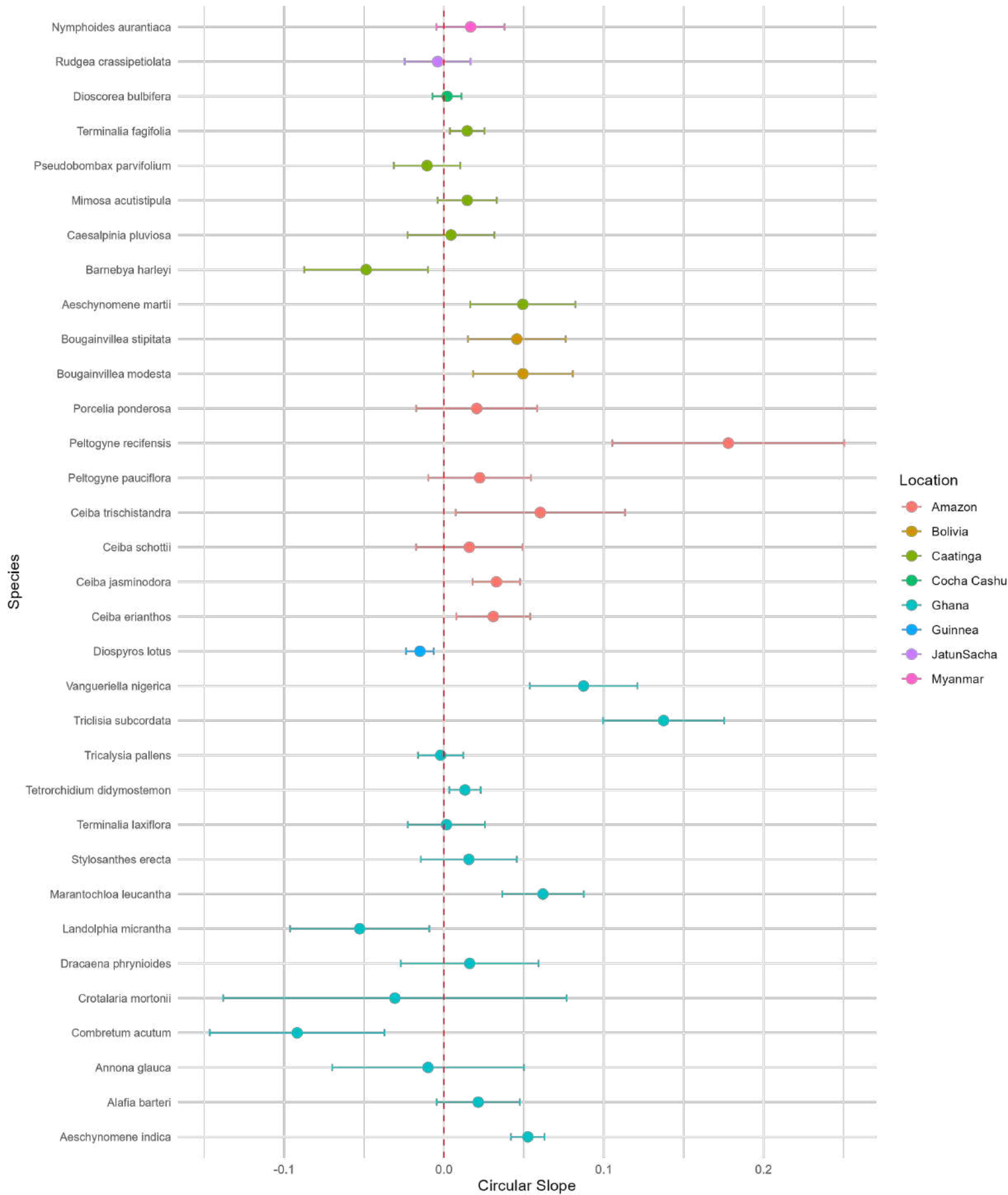

Supplement: S9 Fig — Species arranged by location. (PDF) [file pone.0342105.s011.pdf]

# $\Delta$ DOY/year for One Specimen per Day

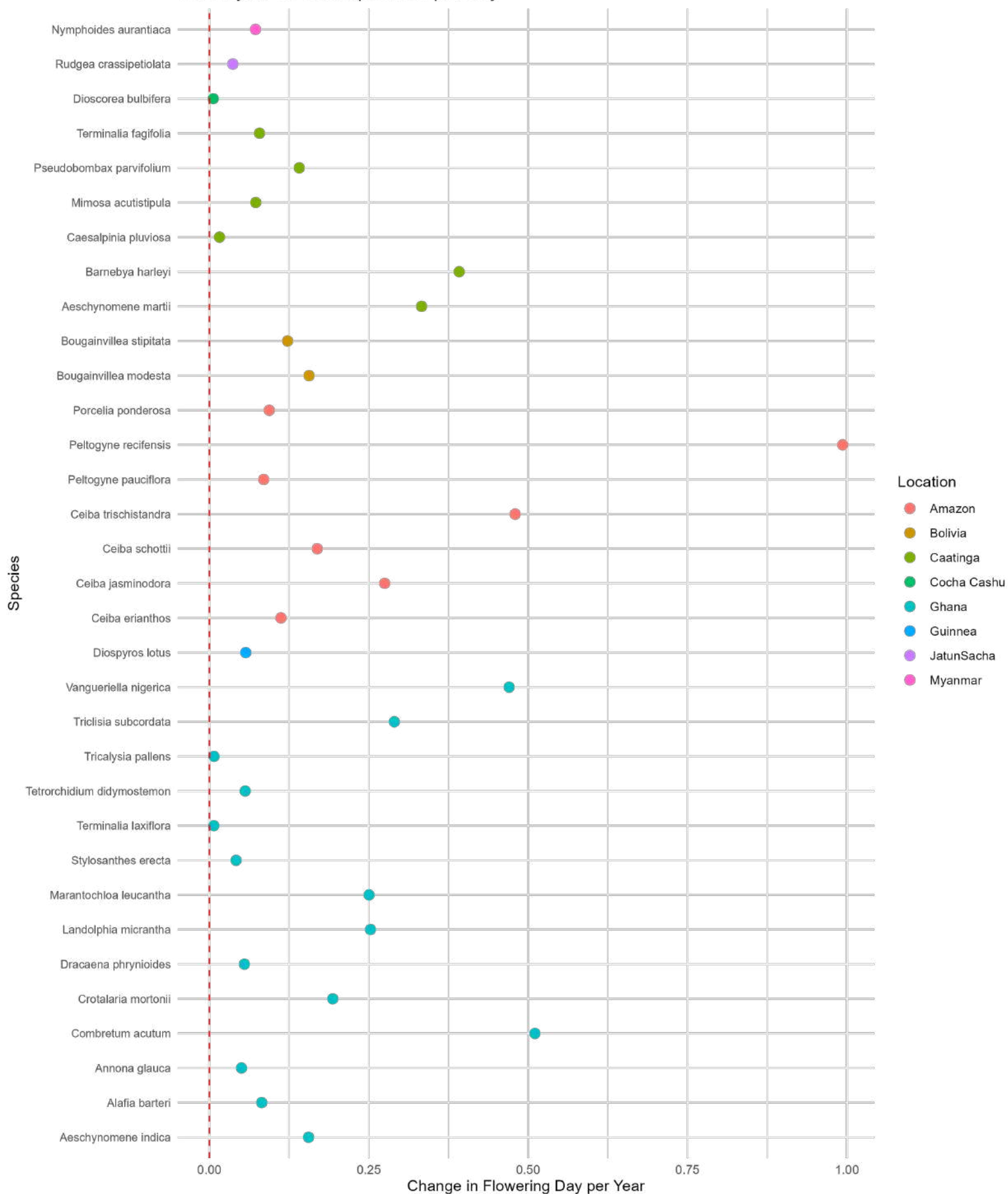

Supplement: S10 Fig — Species arranged by location. (PDF) [file pone.0342105.s012.pdf]
