# Supplementary material for: Observing shifts in phenology of tropical flowering plants
Source: PLoS One. 2026 Feb 25;21(2):e0342105. doi: 10.1371/journal.pone.0342105 (PMC12935240; doi:10.1371/journal.pone.0342105)
Supplement: Attachment — Submitted filename: Response to reviewers.docx [file pone.0342105.s014.docx]

Response to reviewers

We thank the editor and reviewers for taking the time to read and review our submission. We hope we have addressed your concerns thoroughly. Our response is in red.

Journal Requirements:

When submitting your revision, we need you to address these additional requirements.

1. Please ensure that your manuscript meets PLOS ONE's style requirements, including those for file naming. The PLOS ONE style templates can be found at

<https://journals.plos.org/plosone/s/file?id=wjVg/PLOSOne_formatting_sample_main_body.pdf> and [https://journals.plos.org/plosone/s/file?id=ba62/PLOSOne_formatting_sample_title_authors_affiliations.pdf](https://nam10.safelinks.protection.outlook.com/?url=https%3A%2F%2Ftrack.editorialmanager.com%2FCL0%2Fhttps%3A%252F%252Fjournals.plos.org%252Fplosone%252Fs%252Ffile%253Fid%3Dba62%252FPLOSOne_formatting_sample_title_authors_affiliations.pdf%2F1%2F010f019b027f9e44-ae453ed6-5362-4fba-901f-be7314a37f50-000000%2Fv-4PScXLj6vAc7ZDSm7oJSfnH5CpsWGIA7EEMXh0pmM%3D240&data=05%7C02%7CSkylar.Lynch%40colorado.edu%7Cadeedabe5e0c446988d208de37079129%7C3ded8b1b070d462982e4c0b019f46057%7C1%7C1%7C639008702819745019%7CUnknown%7CTWFpbGZsb3d8eyJFbXB0eU1hcGkiOnRydWUsIlYiOiIwLjAuMDAwMCIsIlAiOiJXaW4zMiIsIkFOIjoiTWFpbCIsIldUIjoyfQ%3D%3D%7C0%7C%7C%7C&sdata=sf4dr%2B%2FCSAjMdldFVcm2MEJU3eS7V4du9EucU08F7ug%3D&reserved=0)

Style requirements changed for “Manuscript” file.

2. We note that Figure 1 in your submission contains a map image which may be copyrighted. All PLOS content is published under the Creative Commons Attribution License (CC BY 4.0), which means that the manuscript, images, and Supporting Information files will be freely available online, and any third party is permitted to access, download, copy, distribute, and use these materials in any way, even commercially, with proper attribution. For these reasons, we cannot publish previously copyrighted maps or satellite images created using proprietary data, such as Google software (Google Maps, Street View, and Earth). For more information, see our copyright guidelines: <http://journals.plos.org/plosone/s/licenses-and-copyright>.

We require you to either (1) present written permission from the copyright holder to publish these figures specifically under the CC BY 4.0 license, or (2) remove the figures from your submission:

1. You may seek permission from the original copyright holder of Figure 1 to publish the content specifically under the CC BY 4.0 license.

We recommend that you contact the original copyright holder with the Content Permission Form (http://journals.plos.org/plosone/s/file?id=7c09/content-permission-form.pdf) and the following text:

“I request permission for the open-access journal PLOS ONE to publish XXX under the Creative Commons Attribution License (CCAL) CC BY 4.0 (http://creativecommons.org/licenses/by/4.0/). Please be aware that this license allows unrestricted use and distribution, even commercially, by third parties. Please reply and provide explicit written permission to publish XXX under a CC BY license and complete the attached form.”

Please upload the completed Content Permission Form or other proof of granted permissions as an "Other" file with your submission.

In the figure caption of the copyrighted figure, please include the following text: “Reprinted from [ref] under a CC BY license, with permission from [name of publisher], original copyright [original copyright year].”

2. If you are unable to obtain permission from the original copyright holder to publish these figures under the CC BY 4.0 license or if the copyright holder’s requirements are incompatible with the CC BY 4.0 license, please either i) remove the figure or ii) supply a replacement figure that complies with the CC BY 4.0 license. Please check copyright information on all replacement figures and update the figure caption with source information. If applicable, please specify in the figure caption text when a figure is similar but not identical to the original image and is therefore for illustrative purposes only.

The following resources for replacing copyrighted map figures may be helpful:

USGS National Map Viewer (public domain): <http://viewer.nationalmap.gov/viewer/>
The Gateway to Astronaut Photography of Earth (public domain): <http://eol.jsc.nasa.gov/sseop/clickmap/>
Maps at the CIA (public domain): <https://www.cia.gov/library/publications/the-world-factbook/index.html> and [https://www.cia.gov/library/publications/cia-maps-publications/index.html](https://nam10.safelinks.protection.outlook.com/?url=https%3A%2F%2Ftrack.editorialmanager.com%2FCL0%2Fhttps%3A%252F%252Fwww.cia.gov%252Flibrary%252Fpublications%252Fcia-maps-publications%252Findex.html%2F1%2F010f019b027f9e44-ae453ed6-5362-4fba-901f-be7314a37f50-000000%2FE8yOLf1SUVwlNMfQn1-XDBjn-KuPaqzgq9FHF7S-usw%3D240&data=05%7C02%7CSkylar.Lynch%40colorado.edu%7Cadeedabe5e0c446988d208de37079129%7C3ded8b1b070d462982e4c0b019f46057%7C1%7C1%7C639008702819773316%7CUnknown%7CTWFpbGZsb3d8eyJFbXB0eU1hcGkiOnRydWUsIlYiOiIwLjAuMDAwMCIsIlAiOiJXaW4zMiIsIkFOIjoiTWFpbCIsIldUIjoyfQ%3D%3D%7C0%7C%7C%7C&sdata=9VG6jyKZhRj7k%2BvkrDrswS8so8mCrM447n91QhfxCUs%3D&reserved=0)
NASA Earth Observatory (public domain): <http://earthobservatory.nasa.gov/>
Landsat: <http://landsat.visibleearth.nasa.gov/>
USGS EROS (Earth Resources Observatory and Science (EROS) Center) (public domain): [http://eros.usgs.gov/#](http://eros.usgs.gov/)
Natural Earth (public domain): <http://www.naturalearthdata.com/>

New (non-copyright) map created using R package “maps”.

3. Please include captions for your Supporting Information files at the end of your manuscript, and update any in-text citations to match accordingly. Please see our Supporting Information guidelines for more information: [http://journals.plos.org/plosone/s/supporting-information](https://nam10.safelinks.protection.outlook.com/?url=http%3A%2F%2Ftrack.editorialmanager.com%2FCL0%2Fhttp%3A%252F%252Fjournals.plos.org%252Fplosone%252Fs%252Fsupporting-information%2F1%2F010f019b027f9e44-ae453ed6-5362-4fba-901f-be7314a37f50-000000%2FnBC7Ne5lXFIMI9CThG7OJkzzkNLhvVTAGYd-y5xc4y4%3D240&data=05%7C02%7CSkylar.Lynch%40colorado.edu%7Cadeedabe5e0c446988d208de37079129%7C3ded8b1b070d462982e4c0b019f46057%7C1%7C1%7C639008702819804782%7CUnknown%7CTWFpbGZsb3d8eyJFbXB0eU1hcGkiOnRydWUsIlYiOiIwLjAuMDAwMCIsIlAiOiJXaW4zMiIsIkFOIjoiTWFpbCIsIldUIjoyfQ%3D%3D%7C0%7C%7C%7C&sdata=nI7mcQ9QnlUTsbFfp8MKTkO0EJUafhtmxUbKgO2npfs%3D&reserved=0).

Most supporting information moved to main text. Remaining supporting information updated accordingly.

4. Please upload a new copy of Figure S6 as the detail is not clear. Please follow the link for more information:  <https://journals.plos.org/plosone/s/figures>
 New copy of Figure (now figure 9) uploaded

If the reviewer comments include a recommendation to cite specific previously published works, please review and evaluate these publications to determine whether they are relevant and should be cited. There is no requirement to cite these works unless the editor has indicated otherwise. 


[Note: HTML markup is below. Please do not edit.]

Reviewers' comments:

Reviewer's Responses to Questions

**Comments to the Author**

1. Is the manuscript technically sound, and do the data support the conclusions?

The manuscript must describe a technically sound piece of scientific research with data that supports the conclusions. Experiments must have been conducted rigorously, with appropriate controls, replication, and sample sizes. The conclusions must be drawn appropriately based on the data presented.

Reviewer #1: Partly

Reviewer #2: Yes

2. Has the statistical analysis been performed appropriately and rigorously?

Reviewer #1: No

Reviewer #2: Yes

3. Have the authors made all data underlying the findings in their manuscript fully available?

The [PLOS Data policy](https://nam10.safelinks.protection.outlook.com/?url=http%3A%2F%2Ftrack.editorialmanager.com%2FCL0%2Fhttp%3A%252F%252Fwww.plosone.org%252Fstatic%252Fpolicies.action%2523sharing%2F1%2F010f019b027f9e44-ae453ed6-5362-4fba-901f-be7314a37f50-000000%2F9t7rL8W7pJg16iPRZxsV4WkMIFIcQ4U5hzNDmZpqdzY%3D240&data=05%7C02%7CSkylar.Lynch%40colorado.edu%7Cadeedabe5e0c446988d208de37079129%7C3ded8b1b070d462982e4c0b019f46057%7C1%7C1%7C639008702819829519%7CUnknown%7CTWFpbGZsb3d8eyJFbXB0eU1hcGkiOnRydWUsIlYiOiIwLjAuMDAwMCIsIlAiOiJXaW4zMiIsIkFOIjoiTWFpbCIsIldUIjoyfQ%3D%3D%7C0%7C%7C%7C&sdata=yaqMnCvhLwT8gOIn9xu%2BR%2FKFLyxgjH0m6VcJ0VmucyM%3D&reserved=0) requires authors to make all data underlying the findings described in their manuscript fully available without restriction, with rare exception (please refer to the Data Availability Statement in the manuscript PDF file). The data should be provided as part of the manuscript or its supporting information, or deposited to a public repository. For example, in addition to summary statistics, the data points behind means, medians and variance measures should be available. If there are restrictions on publicly sharing data—e.g. participant privacy or use of data from a third party—those must be specified.

Reviewer #1: Yes

Reviewer #2: Yes

4. Is the manuscript presented in an intelligible fashion and written in standard English?

PLOS ONE does not copyedit accepted manuscripts, so the language in submitted articles must be clear, correct, and unambiguous. Any typographical or grammatical errors should be corrected at revision, so please note any specific errors here.

Reviewer #1: Yes

Reviewer #2: Yes

5. Review Comments to the Author

Please use the space provided to explain your answers to the questions above. You may also include additional comments for the author, including concerns about dual publication, research ethics, or publication ethics. (Please upload your review as an attachment if it exceeds 20,000 characters)

Reviewer #1: Details about responses to the questions above: Some of the data do not support the conclusions, particularly in the comparisons of the findings regarding shifts in flowering phenology in tropical latitudes to findings from other locations. Specifically, more attention needs to be given to the direction of shifts, the uniformity of shift directions among species, and the 'severity' of responses. Given that collection time is proxying for flowering time, attempts to tease out any effects of collection time on the flowering phenological results could be made or explanations could be give for why this may not be possible. The fit of the regression lines used to determine the responses of flowering to time should be analyzed. The criteria for inclusion of specimens in the study should be better justified.

Thank you for your feedback. The inclusion of the tests conducted (previously in the supplemental materials, now in the main text) should address the above concerns. Additional exposition of comparison to non-tropical latitudes added in discussion section. Direction of shifts specified in results and discussion. It has been shown that the unwanted impacts of collector bias can be ameliorated with large datasets and utilization of mean flowering, as opposed to peak or first flowering (25-30 32, 33-34). Additionally, it has been shown that phenology studies with field observations yielded similar results to those utilizing only herbarium specimens (25-30 32, 33-34). Due to the nature of a Bayesian analysis, testing the fit of the regression is built into the process. The training algorithm was a mix of Gibbs sampling, fast rejection sampling, and Metropolis-Hastings. Using two training algorithms is the most efficient and stable way to fit a Bayesian model where no single sampling regime works for all parameters. Four chains were used, with a burnin of 200 and 2500 iterations. The convergence of the chains indicates the best model fit. Furthermore, uncertainty was assessed using standard deviations of the posterior samples for each slope parameter associated with each species. The inclusion of the six data subsets should address the concern regarding the justification for the inclusion of the species.

Abstract
More justification for the potential ecological significance of the study focus rather than justification based solely on the lack of previous studies with this focus would strengthen the overall presentation of the work.

Understood. This change has been made.

The second bullet point cites ‘this hypothesis’. To emphasize the reference, I would suggest changing ‘postulated’ to ‘hypothesized’ in the previous bullet point. Also in the second bullet point, an ‘of’ is missing between ‘phenology flowering’.

Understood. This change has been made.

Use consistency in the presentation of significant digits in the ‘Results’.

Understood. This change has been made.

More ecological context in the ‘Conclusion’ section would strengthen the Abstract.

Understood. This change has been made.

Introduction
Is ‘anthropogenic-mediated’ relevant in the first sentence? Such shifts would apply to climate change more broadly. Also, this sentence needs citation.

Term ‘anthropogenic-mediated’ has been removed and citation has been added.

The ‘cascade effects’ (line 34) could be extrapolated. Cascading impacts on what? I would rewrite this and the following sentence as one: ‘Responses of plants to climate change can have cascading impacts across ecosystems.’

Extrapolation on the cascade effects has been done. Sentences have been re-written as one, as suggested.

The paragraph of lines 40-44 is very short. Could this be further described on combined with the previous paragraph?

Lines have been combined with previous paragraph.

The ‘universally recognized importance’ cited in line 45 needs support.

Citation added.

Some link between the biodiversity information in lines 46-49 and the study focus on phenology should be made. Does this biodiversity suggest the potential for strong cascading effects, for example?

Connecting information added.

What do flowers from plants with discrete flowering periods in the tropics generally respond to if not temperature- or photoperiod-dependent? If not temperature, why would flowering phenology shifts be hypothesized? What proportion of plant species in the tropics have discrete flowering periods? Is this common?

It is not that plants in the tropics don’t respond to temperature, it is that there is not the same distinct seasonality as a cold/dark induced dormancy period that cause either spring or autumn flowering to be most common across taxa. There is more complexity to the triggers of flowering in tropical latitudes. We address this topic in depth in a paper currently in review at PNAS.

The proportion of tropical plants with discrete flowering periods is relatively small, however, species that have discrete flowering periods are more tractable from a perspective of understanding phenological change, as these flowering patterns mirror those seen in non-tropical latitudes. Such species therefore enable comparison to phenological changes documented from non-tropical latitudes. From an ecological perspective, species with discrete flowering periods have a higher likelihood of misaligned interspecific interactions as a result of changes in reproductive period (22, 23). Measuring species with discrete flowering times is therefore likely to target the species most vulnerable to change.

The sentence in lines 54-55 is unnecessary.

Understood. This change has been made.

The paragraph in lines 60-63 is very short. Either elaborate or combine this with the previous paragraph as the topic seems the same. Also, the sentence in lines 60-61 seems overly obvious.

Understood. This change has been made.

What are examples of global impacts that changes in the flowering phenology of tropical plants could have (lines 65-67)?

Examples have been added.

Were the 33 plants selected at random? Or were these the only ones that fit the discrete-flowering criterion for which specimens were available? If the latter, I would use the word ‘identified’ instead.

These were the only ones that fit the discrete-flowering criterion for which specimens were available. Term changed to ‘identified’

The paragraph in lines 71-77 is unnecessary. Limitations of the study should be described in the Discussion section.
Paragraph deleted.

Methods
Remove the first part of the first sentence unless it is made less general.

First part of sentence removed.

Was any analysis of collection efforts through time made to tease out any influence of collection effort changes on flowering phenology shifts? For example, was there no clear pattern of changes in collection efforts?

Rates of collections were analyzed in selection of locations. With the exception of a reduction of collections during the World Wars, there was a steady rate of collections across time, hence the selection of these locations over ones with less frequent/regular collections.

Herbarium collections ‘prior’ to when? (line 82) Also, I would change ‘regularity’ to ‘consistency’.

Terminology changed as requested.

What ‘criteria for inclusion’ are being referenced in line 85?

Please see table 2 and all information moved from supplemental to main text.

‘Mean’ flowering vs. ‘peak’ flowering could be explained in lines 95-96. What was measured in the present study?

Explanation on difference between mean flowering, peak flowering and first flowering added. Mean flowering was used in this study.

The statement in lines 96-98 is attributable to a single study and this should be described. This is not necessarily a broader generalization.

I cited the first study to establish this idea, however I have now since added more studies that support this idea.

Is the first ‘species’ that appears in line 111 meant to be ‘specimens’?

Yes. Apologies for the typo.

Why was 4 or less months/year of flowering considered the cut-off for including species in this study? Why not 5 months or less? Would this have affected the results potentially? Similarly, please justify other inclusion criteria (i.e., 20 specimens per site, 29 years of data, etc.).

All of this was addressed in the supplemental, which has since been moved to the main text. We hope this explains the rationality effectively.

Was there any possibility of making distinctions between flowering stages that could have further informed the timing of flowering phenology?

It has been shown that the unwanted impacts of collector bias can be ameliorated with large datasets and utilization of mean flowering, as opposed to peak or first flowering (25-30 32, 33-34). For this reason, we chose to use Mean flowering. Although we could theoretically look at various phenophases, we would have less confidence in the results, and it would take additional months of manual review of the specimens.

Can some explanation of how collection and flowering generally align be added to strengthen the use of collection time as a proxy for flowering time? Flowering specimens are encouraged in herbarium collections.

This information is added.

Were line regressions used to derive the absolute slopes analyzed for significance (fit of the yearly points along the best-fit line)?

Due to the nature of a Bayesian analysis, testing the fit of the regression is built into the process. The training algorithm was a mix of Gibbs sampling, fast rejection sampling, and Metropolis-Hastings. Using two training algorithms is the most efficient and stable way to fit a Bayesian model where no single sampling regime works for all parameters. Four chains were used, with a burnin of 200 and 2500 iterations. The convergence of the chains indicates the best model fit. Furthermore, uncertainty was assessed using standard deviations of the posterior samples for each slope parameter associated with each species.

Results
Changes in flowering date should be described in terms of earlier vs. later? A change in and of itself is not as informative. I would present results for the negative sloped species distinctly from the positively sloped species as these could represent distinct ecological responses that could have distinct ecological relevance.

Thank you, this change has been made.

The figure captions should be more ‘stand-alone’ by explaining abbreviations used, study focus on the tropics, etc.

Thank you, this change has been made.

In the figures, could species be presented by family vs. location?

In theory, yes, but we believe the representation by location is a more important grouping. We hope the inclusion of the phylogeny/ Bloomberg’s K fulfills the desire to see the phylogenetic patterns in the data.

Suggest moving Table 2 to the Supplement. Some of the information in this table is double depicted in the figures.

Thank you, this change has been made.

Discussion
The study brings new ‘information’ to light, not new ‘data’.

Thank you, this change has been made.

In non-tropical latitudes, are shifts more likely to be in one direction (i.e., earlier in the spring, later in the fall)? Or do you similar see changes in both directions depending on the species and location?

Information addressing this question added ” Although there is not strict uniformity in the direction of change in flowering date in non-tropical locations, there is a greater consistency, due in large part to the drivers of flowering, such as increase or decrease in temperature and photoperiod (28, 47 -52).”

The assertion that tropical shifts are not ‘less severe’ than those documented at other latitudes needs to be substantiated?

Additional comparison to non-tropical studies added.

How ‘severe’ were those shifts? The existence of shifts and their severity are distinct concepts (lines 173-177).
Terminology changed.

Why was your prediction that the species would show a lack of uniformity in the direction of their flowering phenology shifts (line 177, lines 196-198)?

Information added on rational for prediction. Reason is that there is not the same distinct seasonality as a cold/dark induced dormancy period that cause either spring or autumn flowering to be most common across taxa. There is more complexity to the triggers of flowering in tropical latitudes. We address this topic in depth in a paper currently in review at PNAS.

Lines 182-192: Again, the direction of the shifts seems ecological relevant. Direction should be discussed.
Thank you, this change has been made.

If precipitation is suspected to be a major driver of phenological shifts in the tropics, could that have been investigated here?

We have dedicated an entire paper to this question. It is currently in review at PNAS.

What major changes in precipitation have occurred through time in the study locations? Could that explain phenological shifts in flowering?

We have dedicated an entire paper to this question. It is currently in review at PNAS.

A major limitation of the Discussion is the lack of attention given to the ecological relevance of the findings. What could the detected shifts (which were not uniform in direction) mean for other species, communities, ecosystem? How pollinator-specific are these species (i.e., are they generalists or specialists in this regard)? Are they all primary animal-pollinated? Have other systems at other latitudes also showed such non-uniformity in the direction of phenological shifts, and if so, what has that meant for other species, communities, ecosystems?

A table has been added addressing the pollinators, seed dispersers, habit and IUCN Red List Status of each species. Multiple paragraphs added throughout addressing the biological implications.

The ’Human Impacts’ subsection seems extraneous. More important would be discussion of the potential ecological relevance of the findings.

Paragraph removed and replaced with one addressing the ecological implications.

Reviewer #2: In PONE-D-25-54215 entitled “Observing shifts in phenology of tropical flowering plants”, the authors test for phenological shifts in tropical flowering plants. They were particluarlly interested in comparing their results with temperate species and thus restricted their analyses to species with distinct seasonal patterns (i.e., blooming for 3-4 months). The manuscript was very easy to read and addressed a timely topic that would be of interest to a broad audience. Many of the species observed exhibited phenological shifts to those documented for temperate species, which has interesting ecological implications. I commend the authors for their significant efforts to ensure robust results and to discuss them without hyperbole. However, there are several parts of the manuscript that would benefit from increased clarity and depth (see my comments below for details). Upon revision, I think this manuscript will make an important contribution to the field.

Thank you for your kind words! 😊

While easy to read, the manuscript appeared to lack sufficient support for the claims upon first reading. However, this was because much of the pertinent information is in the supplementary materials and never referenced in the main text. The authors conducted several analyses to test potential biases or weaknesses in their analyses. These additional analyses indicate that their results are robust to those biases, but they were not described or referenced in the main text as they should be (indeed it would strengthen the paper to include them).

All material in the supplemental materials has been moved to the main text to address these concerns.

Moreover, two results deserve additional discussion. The first is the similar pacing of phenological change before and after 1960. Given that climate change is accelerating, seeing similar changes in both time periods could indicate a slower rate of response or constraints on phenological shifts. While the authors do not directly test that idea, it warrants further discussion in the main text. The presence of a phylogenetic signal, while not particularly strong, also deserves discussion in the main text.

Although the pacing of phenological change is similar before and after 1960, it is not the same, and we are seeing an increase in rate of change in relation to the increase in rate of climate change. The phylogenetic tests and results have been moved to the main text.

As is common in phenology studies, species varied in the degree of change through time. However, the authors provide little rationale for why we might see such differences in this data set. What are the documented climate change patterns for these locations? What habitats are represented? In which habitats are the species found and do they specialize on specific habitats? Are these long-lived or short-lived species? What kinds of growth habits do they exhibit (e.g., forbs, woody vines or shrubs, trees)? All of these factors could influence species' response to climate changes, and it is unclear what subset of tropical habits were studied here. I am not suggesting that the authors have to change their analyses to test whether any of these factors predict phenological changes. However, these traits do warrant description in the methods and, if there are indicators that any of these factors might influence species responses, they should be addressed in the discussion.

A table has been added addressing the pollinators, seed dispersers, habit and IUCN Red List Status of each species.

Finally, the methods need more detail for them to be interpretable, particularly for those less familiar with circular linear regression (e.g., description of the purpose of the training algorithm and the various sampling regimes).

Additional clarification to the purpose of the training algorithm and the various sampling regimes has been added. If this is insufficient I request more specifics on what exactly needs more detail so that I may best address your concerns.

6. PLOS authors have the option to publish the peer review history of their article ([what does this mean?](https://nam10.safelinks.protection.outlook.com/?url=https%3A%2F%2Ftrack.editorialmanager.com%2FCL0%2Fhttps%3A%252F%252Fjournals.plos.org%252Fplosone%252Fs%252Feditorial-and-peer-review-process%2523loc-peer-review-history%2F1%2F010f019b027f9e44-ae453ed6-5362-4fba-901f-be7314a37f50-000000%2FkduraCpteOPyELEdp6VUVPoRwFPu1xQm56jaMMh87zs%3D240&data=05%7C02%7CSkylar.Lynch%40colorado.edu%7Cadeedabe5e0c446988d208de37079129%7C3ded8b1b070d462982e4c0b019f46057%7C1%7C1%7C639008702819851742%7CUnknown%7CTWFpbGZsb3d8eyJFbXB0eU1hcGkiOnRydWUsIlYiOiIwLjAuMDAwMCIsIlAiOiJXaW4zMiIsIkFOIjoiTWFpbCIsIldUIjoyfQ%3D%3D%7C0%7C%7C%7C&sdata=dzmhr4rsDhCMs47c2DAvZr1DMyp29y%2FGjHyzl78T9kA%3D&reserved=0)). If published, this will include your full peer review and any attached files.

If you choose “no”, your identity will remain anonymous but your review may still be made public.

**Do you want your identity to be public for this peer review?** For information about this choice, including consent withdrawal, please see our [Privacy Policy](https://nam10.safelinks.protection.outlook.com/?url=https%3A%2F%2Ftrack.editorialmanager.com%2FCL0%2Fhttps%3A%252F%252Fwww.plos.org%252Fprivacy-policy%2F1%2F010f019b027f9e44-ae453ed6-5362-4fba-901f-be7314a37f50-000000%2F_vqcA8DqzBJPXw-aq2pyrOjJEHbGE6cQlRAsikoJ1yc%3D240&data=05%7C02%7CSkylar.Lynch%40colorado.edu%7Cadeedabe5e0c446988d208de37079129%7C3ded8b1b070d462982e4c0b019f46057%7C1%7C1%7C639008702819878373%7CUnknown%7CTWFpbGZsb3d8eyJFbXB0eU1hcGkiOnRydWUsIlYiOiIwLjAuMDAwMCIsIlAiOiJXaW4zMiIsIkFOIjoiTWFpbCIsIldUIjoyfQ%3D%3D%7C0%7C%7C%7C&sdata=7KNKvJ7%2FmOXfNwG3z45HAkpYv95mqBtsiyZNui9psSg%3D&reserved=0).

Reviewer #1: No

Reviewer #2: No

[NOTE: If reviewer comments were submitted as an attachment file, they will be attached to this email and accessible via the submission site. Please log into your account, locate the manuscript record, and check for the action link "View Attachments". If this link does not appear, there are no attachment files.]

To ensure your figures meet our technical requirements, please review our figure guidelines: <https://journals.plos.org/plosone/s/figures>

You may also use PLOS’s free figure tool, NAAS, to help you prepare publication quality figures: <https://journals.plos.org/plosone/s/figures#loc-tools-for-figure-preparation>.

NAAS will assess whether your figures meet our technical requirements by comparing each figure against our figure specifications.

*In compliance with data protection regulations, you may request that we remove your personal registration details at any time.* [*(Remove my information/details)*](https://nam10.safelinks.protection.outlook.com/?url=https%3A%2F%2Ftrack.editorialmanager.com%2FCL0%2Fhttps%3A%252F%252Fwww.editorialmanager.com%252Fpone%252Flogin.asp%253Fa%3Dr%2F1%2F010f019b027f9e44-ae453ed6-5362-4fba-901f-be7314a37f50-000000%2F5v5BTxHEs7iv0TYF-3FIyEYXtvX4F8tTYYDezbmfAUA%3D240&data=05%7C02%7CSkylar.Lynch%40colorado.edu%7Cadeedabe5e0c446988d208de37079129%7C3ded8b1b070d462982e4c0b019f46057%7C1%7C1%7C639008702819900337%7CUnknown%7CTWFpbGZsb3d8eyJFbXB0eU1hcGkiOnRydWUsIlYiOiIwLjAuMDAwMCIsIlAiOiJXaW4zMiIsIkFOIjoiTWFpbCIsIldUIjoyfQ%3D%3D%7C0%7C%7C%7C&sdata=hC7KNix5QUl8HT19KwAz9wD0f1qjoRNLjI%2BoABPVAfU%3D&reserved=0)*. Please contact the publication office if you have any questions.*
