# Supplementary material for: Observing shifts in phenology of tropical flowering plants
Source: PLoS One. 2026 Feb 25;21(2):e0342105. doi: 10.1371/journal.pone.0342105 (PMC12935240; doi:10.1371/journal.pone.0342105)
Supplement: Attachment — Submitted filename: Response to reviewers_2026.docx [file pone.0342105.s015.docx]

Below are the reviewers comments in red and my responses in black.

The author thoroughly responded to my recommendations (at times may have even over-corrected). I appreciate all of the changes the authors made to the text and tables but suggest that some of the added figures are unnecessary. Presenting the results of the competing models in table 3 was sufficient to address my concerns regarding providing evidence to support their claims in the text. In my opinion, Figures 4-8 could remain in the supplementary materials.

I am glad you found the corrections thorough! Figures 4-8 have been returned to the supplementary materials.

I would be interested in seeing the phylogeny with the change in flowering mapped on (color-coded by slope or similar would be sufficient). At present, the reader must cross reference multiple figures in order to connect the phylogeny in the figure with the stated results. If the response was mapped onto the phylogeny, the phylogenetic patterns would be much more interpretable.

I made a phylogeny color coded by the change in flowering date in days.

Because the figure panels were each uploaded separately, the scaling of the text will be off once the panels are combined into a single figure, making the labels challenging to read. The authors will likely need to modify the figures to make them more legible.

I believe this is referring to the 2 paneled figures of figures 4-8. Because these have been moved to the supplement, I simplified by making each figure independent instead of paneled. I split the figure captions to match.

The “Outlier Species” section should be included in the “Directionality of change...” section. In my opinion, it doesn’t warrant its own section.

I moved the “Outlier Species” section to the “Directionality of change” section

Discussing how these analyses were generally robust to potential biases would be informative for the field. However, the length of the section “Impacts of implementation of dataset criteria” could be shortened to include only the first 2 paragraphs which synthesize the patterns rather than going through each criterion in detail.

I shortened the section to only include the first two paragraphs as suggested.

Overall, I commend the authors for their thorough changes and look forward to seeing the final product in print.

Thank you for all your comments and suggestions. I really appreciate the attention to detail you put in to make this paper the best it can be!
